# NAD^+^-Carrying Mesoporous Silica Nanoparticles Can Prevent Oxidative Stress-Induced Energy Failures of Both Rodent Astrocytes and PC12 Cells

**DOI:** 10.1371/journal.pone.0074100

**Published:** 2013-09-09

**Authors:** Heyu Chen, Yao Wang, Jixi Zhang, Yingxin Ma, Caixia Wang, Ying Zhou, Hongchen Gu, Weihai Ying

**Affiliations:** School of Biomedical Engineering and Med-X Research Institute, Shanghai Jiao Tong University, Shanghai, P.R. China; Universidad de Castilla-La Mancha, Spain

## Abstract

**Aim:**

To test the hypothesis that NAD^+^-carrying mesoporous silica nanoparticles (M-MSNs@NAD+) can effectively deliver NAD^+^ into cells to produce cytoprotective effects.

**Methods & Materials:**

NAD^+^ was incorporated into M-MSNs. Primary rat astrocyte cultures and PC12 cells were treated with H_2_O_2_, followed by post-treatment with M-MSNs@NAD+. After various durations of the post-treatment, intracellular NAD^+^ levels, intracellular ATP levels and lactate dehydrogenase (LDH) release were determined.

**Results & Discussion:**

M-MSNs can be effectively loaded with NAD^+^. The M-MSNs@NAD+ can significantly attenuate H_2_O_2_-induced NAD^+^ and ATP decreases in both astrocyte cultures and PC12 cells. M-MSNs@NAD+ can also partially prevent the H_2_O_2_-induced LDH release from both astrocyte cultures and PC12 cells. In contrast, the NAD^+^ that is spontaneously released from the M-MSNs@NAD+ is insufficient to prevent the H_2_O_2_-induced damage.

**Conclusions:**

Our study has suggested the first approach that can effectively deliver NAD^+^ into cells, which provides an important basis both for elucidating the roles of intracellular NAD^+^ in biological functions and for therapeutic applications of NAD^+^. Our study has also provided the first direct evidence demonstrating a key role of NAD^+^ depletion in oxidative stress-induced ATP decreases.

## Introduction

A number of studies have suggested that NAD^+^ plays important roles in a variety of biological processes, such as energy metabolism, mitochondrial functions, calcium homeostasis and aging [1]. It has also been found that NAD^+^ treatment can decrease genotoxic agent-induced death of primary cultures of astrocytes and neurons [2], [3]. Our previous studies have found that NAD^+^ administration can reduce the brain injury in the animal models of cerebral ischemia and traumatic brain injury [4], [5], [6].

It has become increasingly important to elucidate the mechanisms underlying the roles of intracellular NAD^+^ in both biological functions and cell death. However, there have been no approaches that can effectively deliver NAD^+^ into cells without producing confounding effects resulting from NAD^+^-dependent ecto-enzymes, which is the major obstacle for the studies on the roles of intracellular NAD^+^ in biological functions and cell death. The currently used approach for increasing intracellular NAD^+^ concentrations is direct additions of NAD^+^ into cell culture media. Because there are such NAD^+^-dependent ecto-enzymes as CD38 and mono(ADP-ribosyl) transferases on plasma membranes [7], the extracellularly administered NAD^+^ may produce its effects partially by interacting with the ecto-enzymes, which would prevent solid elucidation of the roles of intracellular NAD^+^ in biological functions. Moreover, in the currently used approach for increasing intracellular NAD^+^ concentrations, high concentrations of NAD^+^ (over 1 mM) are required to produce protective effects against genotoxic insults [2], [3]. Therefore, it becomes increasingly important to develop novel approaches to effectively deliver NAD^+^ into cells, which may not only greatly enhance our capacity to solidly elucidate the biological functions of intracellular NAD^+^, but also increase therapeutic potential of NAD^+^.

Magnetic mesoporous silica nanoparticles (M-MSNs) have shown great potential as a multi-functional drug carrier [8], [9], [10], which can be internalized into cells by endocytosis [11]. In this study we determined if M-MSNs may be developed into a NAD^+^ carrier that can effectively deliver NAD^+^ into cells. Our study has suggested that M-MSNs can be effectively loaded with NAD^+^, which can efficiently deliver NAD^+^ into both H_2_O_2_-treated astrocytes and H_2_O_2_-treated PC12 cells to restore the intracellular NAD^+^ and ATP levels of the cells.

## Materials and Methods

### Materials

Cetyltrimethylammonium bromide (CTAB) was purchased from Fluka. Tetraethyl orthosilicate (TEOS), ammonium nitrate (NH_4_NO_3_), and cyclohexane were purchased from Aladdin. Ethyl acetate, absolute ethanol and methanol were purchased from Sinopharm Chemical Reagent Co., Ltd., China. All these reagents are analytical reagent grade. All of the other chemicals were purchased from Sigma (St. Louis, MO, USA) except where noted. Millipore water (18.2 MΩ·cm) was used in the preparations of all aqueous solutions.

### Synthesis of Magnetic Mesoporous Silica Nanoparticles (M-MSNs)

M-MSNs were synthesized according to our previous reports [12]: Uniform magnetic Fe_3_O_4_ nanoparticles (MNPs) stabilized in oleic acid were prepared by the co-precipitation method [13]. The concentration of MNPs dispersed in chloroform was adjusted to 6.0 mg Fe/mL, and subsequently 0.74 mL suspension was added into 5 mL CTAB aqueous solution (0.08 M). After continuous ultrasonication for 30 min at 50°C, a homogeneous oil-in-water emulsion was derived from the mixture above, accompanied with the evaporation of chloroform. Subsequently, another stirring at 70°C for 10 min was performed to evaporate the residual chloroform, resulting in a transparent black dispersion, which indicates that the MNPs were transferred to aqueous phase successfully with the aid of CTAB. The obtained dispersion was diluted with 45 mL water, and subsequently 0.3 mL NaOH solution (2 M), 0.5 mL TEOS and 3 mL ethyl acetate were introduced to the reaction solution sequentially. All of the reagents were stirred at 70°C with refluxing for 3 hrs. The resultant products were collected by centrifugation and washed with ethanol and water three times. Finally, CTAB was removed by a highly efficient ion-exchange method. The purified nanoparticles were dispersed in 60 mL ethanol solution containing 60 mg NH_4_NO_3_ and ultrasonicated in a water bath for 2 hrs, and the procedure was repeated three times to obtain the M-MSNs in which surfactant had been completely removed.

### Loading of M-MSNs with NAD^+^

For loading NAD^+^, 20 mg M-MSNs were suspended into 20 mL cyclohexane solution of NAD^+^ (150 µg/mL). After ultrasonication for 5 min, the mixture was shaken at 25°C for 2 hrs to ensure that the adsorption process could be carried out sufficiently, and separated by centrifugation to collect the NAD^+^-loaded M-MSNs (M-MSNs@NAD^+^). The remaining solvent was removed from particles by drying at 37°C for at least 3 hrs. To determine the absorbed amount of NAD^+^, an indirect eluting method according to previous literatures [14], [15],was performed as follows: Three mg M-MSNs@NAD^+^ were dispersed in 2 mL methanol and sonicated for 10 min. After centrifugated separation, the eluting solution was measured by UV-Vis adsorption at a wavelength of 260 nm to calculate the concentrations of NAD^+^.

### Characterization of M-MSNs

Transmission electron microscopy (TEM) images were recorded on a JEM 2010 (JEOL, Japan) instrument with 200 kV accelerated voltage. Scanning electron microscopy (SEM) images were obtained by a JSM-6460 (JEOL, Japan) microscope operating at 20 kV. Dynamic light scattering (DLS) measurements were performed on a Zetasizer Nano instrument (Malvern, UK) at 298 K to analyze the hydrodynamic diameters of M-MSNs. Nitrogen sorption isotherm was measured at 77 K with a Micromeritcs ASAP2010 analyzer (USA). The concentrations of NAD^+^ were detected on a NanoDrop 1000 spectrophotometer (Thermo Scientific, USA).

### Assays of NAD^+^ Release from M-MSNs@NAD^+^

To study the profile of NAD^+^ release from the M-MSNs@NAD^+^, 10 mg M-MSNs@NAD^+^ were dispersed in 2 mL of phosphate-buffered saline (PBS, pH = 7.4), and the dispersion was transferred into a dialysis bag with a molecular weight cut off of 1000 Da. The dialysis was then kept in 18 mL PBS at 37°C and shaken at a speed of 150 rpm. At different time intervals, 0.2 mL of the solution was collected to test the amount of released NAD^+^ by UV-Vis. To keep constant volume, 0.2 mL of fresh medium was added after each sampling. All NAD^+^ release results were averaged with three measurements.

### Cell Cultures

This protocol was approved by the institutional Animal Care and Use Committee, School of Biomedical Engineering, Shanghai Jiao Tong University, Shanghai, China. All efforts were made to minimize suffering of animals. Primary rat cortical astrocyte cultures were prepared as described previously [16]. In brief, cortices were harvested from 1-day-old mice (SLAC, Shanghai, China). The cortices were dissociated in trypsin, plated in 24-well tissue culture plates in Dulbecco’s Modified Eagle Medium containing 4,500 mg/L D-glucose, 584 mg/L L-glutamine, 110 mg/L sodium pyruvate (Thermo Scientific, Waltham, MA, USA), 1% penicillin and streptomycin (Invitrogen, Carlsbad, CA, USA), and 10% fetal bovine serum (GIBCO, Melbourne, Austria), and maintained at 37°C in a 5% CO_2_ incubator. The cell cultures became confluent 12 to 15 days after the plating, which were treated for 48 h with cytosine arabinoside (10 µm). The cultures could be used two days after removal of cytosine arabinoside.

Differentiated PC12 cells were purchased from the Cell Resource Center of Shanghai Institute of Biological Sciences, Chinese Academy of Sciences, which were plated onto 24-well cell culture plates at the initial density of 1×10^5^ cells/mL, and cultured in the same conditions as the astrocytes.

### Treatment of the Cells with M-MSNs@NAD^+^

M-MSNs and M-MSNs@NAD^+^ suspensions were sonicated in 25 mM HEPES solution (pH 7.2) at concentration of 1 mg/mL, and then diluted in DMEM at the concentrations of 50 µg/mL or 250 µg/mL before use. The cell cultures were treated with H_2_O_2_ for 1 hr, and then washed out and replaced by M-MSN or M-MSNs@NAD^+^ suspensions.

### Lactate Dehydrogenase (LDH) Assay

As described previously [17], cell survival was quantified by measuring LDH activity in culture media. Briefly, 100 µL culture media were mixed with 150 µL 500 mM potassium phosphate buffer (pH 7.5) containing 1.5 mM NADH and 7.5 mM sodium pyruvate, and the A_340 nm_ change was monitored over 90 s. LDH release was calculated by normalizing the LDH values of samples to LDH activity measured in culture media from control culture wells.

### NAD^+^ Assay

NAD^+^ concentrations were measured by recycling assay as previously described [18]. Briefly, samples were extracted in 0.5 N perchloric acid. After centrifugation at 12,000 RPM for 5 min, the supernatant was neutralized to pH 7.2 using 3 N potassium hydroxide and 1 M potassium phosphate buffer. After centrifugation at 12,000 RPM for 5 min, the supernatants were mixed with a reaction medium containing 1.7 mg 3-[4,5-dimethylthiazol-2-yl]-2,5-diphenyl-tetrazolium bromide (MTT), 10.6 mg phenazine methosulfate, 1.3 mg alcohol dehydrogenase, 488.4 mg nicotinamide, and 2.4 mL ethanol in 37.6 mL Gly-Gly buffer (65 mM, pH 7.4). The A_560 nm_ was determined immediately and after 10 min, and the readings were calibrated with NAD^+^ standards. Results were normalized to protein contents as determined by the BCA assay.

### ATP Assay

ATP levels were quantified using the Roche ATP Bioluminescence Assay Kit (HS II) following the standard protocol provided by the vendor. In brief, the cells were washed once with PBS and lysed with the Cell Lysis Reagent. Then 50 µL of the lysates was mixed with 50 µL of the Luciferase Reagent, and the luminescence was detected using a plate reader (Biotek Synergy 2). The protein concentrations of the samples were determined using the BCA assay. The ATP concentrations of the sample were calculated using an ATP standard, and normalized to the protein of the samples.

### Statistical Analyses

All data are presented as mean±SE. Data were assessed by one-way ANOVA, followed by Student-Newman-Keuls *post hoc* test. *P* values less than 0.05 were considered statistically significant.

## Results

### Characterization of the M-MSNs

**Figure 1:**
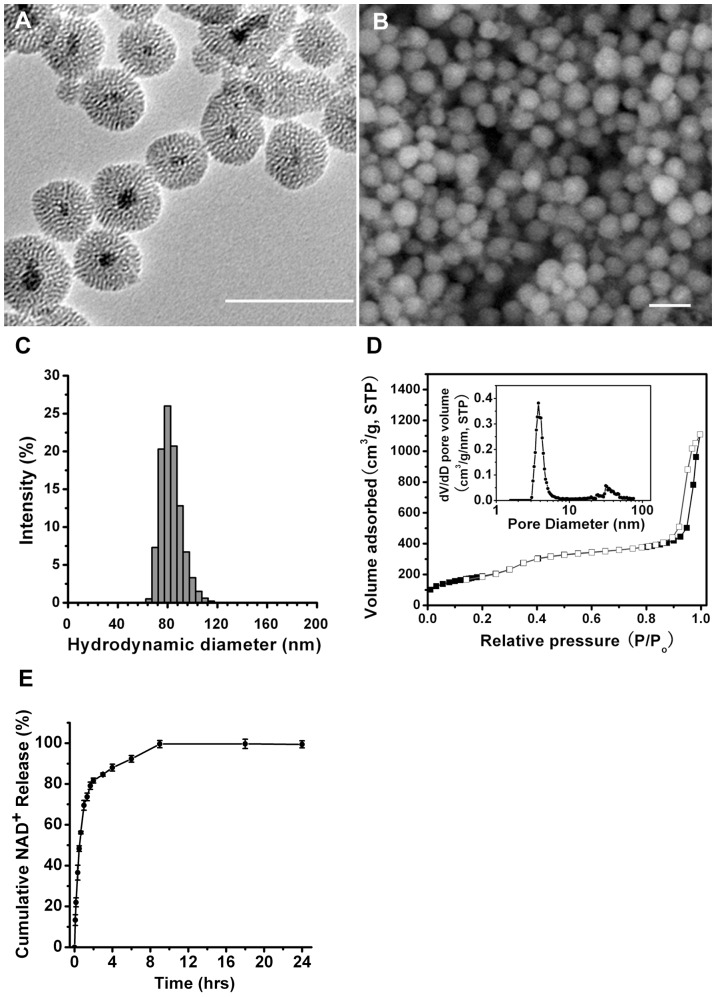
Characterization of the M-MSNs. Representative (A) TEM image and (B) SEM image of synthesized M-MSNs. Bar = 100 nm. (C) Hydrodynamic diameter distribution of M-MSNs in water. (D) Nitrogen sorption isotherm at 77 K (the inset showed the corresponding pore size distribution). (E) Profile of NAD^+^ release from the M-MSNs@NAD^+^ dissolved in PBS (pH = 7.4) at 37°C. N = 6. Data were collected from two independent experiments.

The M-MSNs prepared by the approach stated above showed a uniform and discrete spherical shape with a mean particle diameter of 80±20 nm, which can be observed obviously in their representative TEM image (Fig. 1A) or SEM image (Fig. 1B). Most M-MSNs possess a typical core-shell composite structure, with a single Fe_3_O_4_ nanocrystal embedded in the center. The mesoporous silica shells of M-MSNs own wormhole-like mesopores that are arranged radially to the surface. The center-located magnetic core has an average size of approximately 15 nm as estimated from Fig. 1A. In addition, the hydrodynamic diameter distribution of M-MSNs in water has a single peak centered at 80 nm, as shown in Fig. 1C. A typical nitrogen isotherm measurement for M-MSNs exhibits a type IV isotherm with the H1 hysteresis loop, according to the International Union of Pure and Applied Chemistry (IUPAC) nomenclature (Fig. 1D). The obvious nitrogen condensation step at the relative pressure P/P_0_ = 0.25–0.4 is reflected in a narrow pore size distribution curve (Fig. 1D, inset) with a sharp peak centered at 3.8 nm, which supports the observed pore structure in Fig. 1A. At high relative pressure regions (P/P_0_>0.9), a secondary condensation step occurred, which should be ascribed to the interparticle spaces formed among accumulative M-MSNs. The surface area and pore volume of M-MSNs were determined to be 696 m^2^/g and 0.44 cm^3^/g, respectively, indicating their high potential in loading sufficient amount of drugs or other bio-molecules.

### NAD^+^ Loading Strategy

NAD^+^ is a hydrophilic molecule with four hydroxyl groups which could form hydrogen bonds with the numerous Si-OH on the inner/outer surfaces of M-MSNs. Considering the basic principle of drug adsorption [19], we have chosen the nonpolar organic solvent cyclohexane to minimize the competing interactions between the solvent and either the adsorbent (M-MSNs) or absorbate (NAD^+^). The loading amount of NAD^+^ with M-MSNs, which was detected by the eluting method mentioned above, was 146.5 µg/mg, indicating that almost all of the NAD^+^ in the initial solution was loaded into the nanoparticles, which should result from the non-polar solvent strategy. We also studied the time course of NAD^+^ release from the M-MSNs@NAD+ dissolved in PBS (pH = 7.4) at 37°C, showing that NAD^+^ was rapidly released within 2 hrs after the M-MSNs@NAD+ was prepared in PBS (Fig. 1E).

### M-MSNs@NAD^+^ can Restore Both the Intracellular NAD^+^ and ATP Levels of H_2_O_2_-treated PC12 Cells

**Figure 2:**
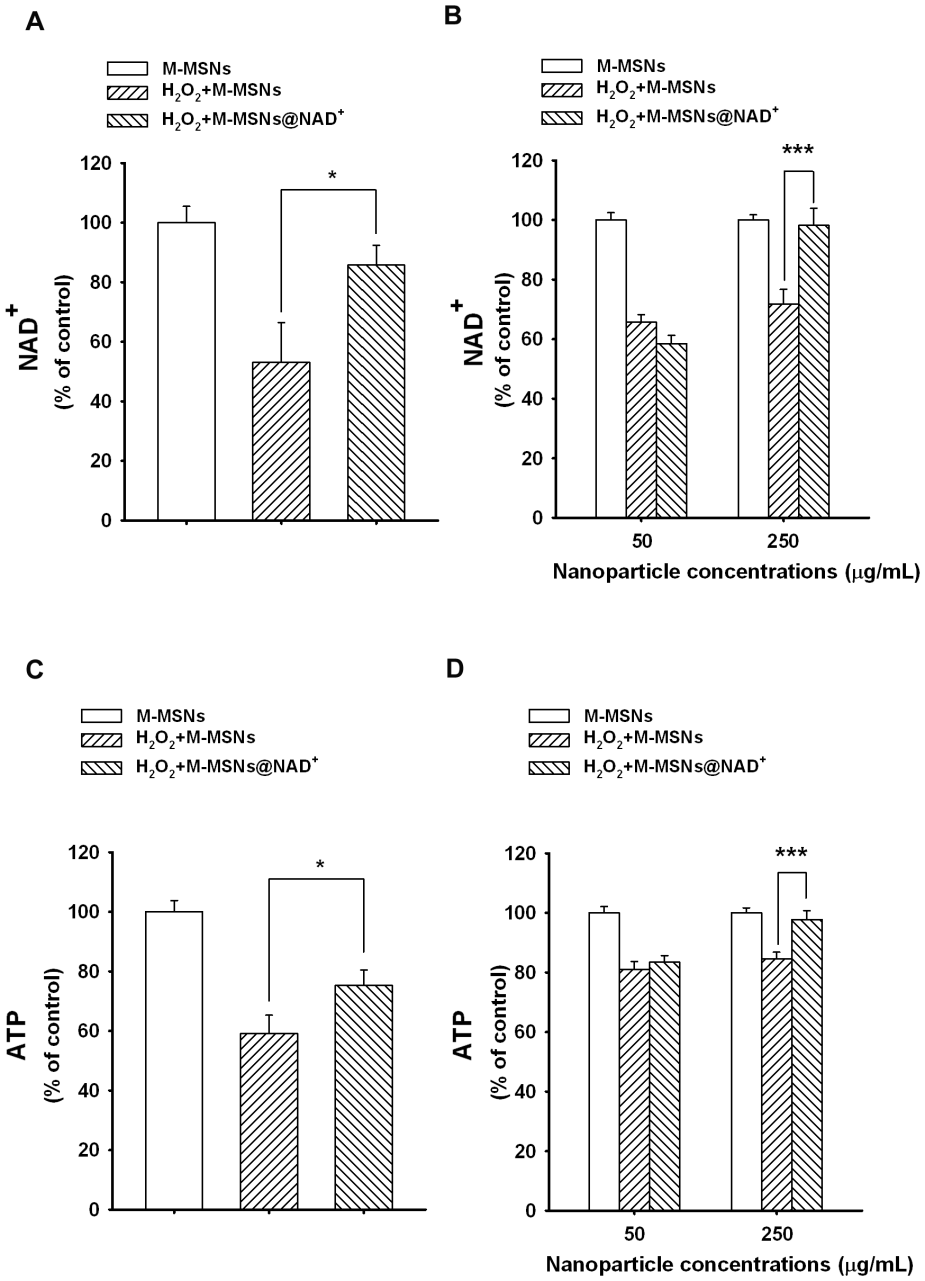
M-MSNs@NAD^+^ can significantly increase the intracellular NAD^+^ and ATP levels of H_2_O_2_-treated PC12 cells. (A) Treatment of the cells with 1 mM H_2_O_2_ led to a significant decrease in the intracellular NAD^+^ levels of PC12 cells, which was largely restored by post-treatment of the cells with 50 µg/mL M-MSNs@NAD^+^ for 3 hrs. N = 29. Data were collected from six independent experiments. *, *p*<0.05. (B) Assessed at 6 hours after the nanoparticle post-treatment, 250 µg/mL M-MSNs@NAD^+^, but not 50 µg/mL M-MSNs@NAD^+^, was shown to prevent the H_2_O_2_-induced decrease in the intracellular NAD^+^ levels of PC12 cells. N = 11∼12. Data were collected from three independent experiments. ***, *p*<0.001. (C) Treatment of the cells with 1 mM H_2_O_2_ also led to a significant decrease in the intracellular ATP levels of PC12 cells, which was also largely restored by post-treatment of the cells with 50 µg/mL M-MSNs@NAD^+^ for 3 hrs. N = 15. Data were collected from three independent experiments. *, *p*<0.05. (D) Assessed at 6 hours after nanoparticle post-treatment, 250 µg/mL M-MSNs@NAD^+^, but not 50 µg/mL M-MSNs@NAD^+^, was capable of preventing the H_2_O_2_-induced decrease in the intracellular ATP levels of PC12 cells. N = 12. Data were collected from three independent experiments. ***, *p*<0.001.

To determine the capacity of the M-MSNs@NAD^+^ to increase intracellular NAD^+^ levels and NAD^+^-dependent cellular functions, we used H_2_O_2_-treated PC12 cells as a cellular model. Treatment of PC12 cells with 1 mM H_2_O_2_ significantly decreased the intracellular NAD^+^ level of the cells (Fig. 2A), which was significantly improved by post-treatment of the cells with the 50 µg/mL M-MSNs@NAD^+^ for 3 hrs (Fig. 2A). We further determined the effects of M-MSNs@NAD^+^ on the intracellular NAD^+^ levels of H_2_O_2_-treated cell at 6 hrs after the M-MSNs@NAD^+^ treatment, showing that 250 µg/mL M-MSNs@NAD^+^, but not 50 µg/mL M-MSNs@NAD^+^, was capable of significantly attenuating the H_2_O_2_-induced decrease in the intracellular NAD^+^ levels of PC12 cells (Fig. 2B).

Treatment of PC12 cells with 1 mM H_2_O_2_ also led to a significant decrease in the intracellular ATP level (Fig. 2C), which was partially restored by post-treatment of the cells with 50 µg/mL M-MSNs@NAD^+^ for 3 hrs (Fig. 2C). We also determined the effects of M-MSNs@NAD^+^ on the intracellular ATP levels of H_2_O_2_-treated cell at 6 hrs after the M-MSNs@NAD^+^ treatment, showing that 250 µg/mL M-MSNs@NAD^+^, but not 50 µg/mL M-MSNs@NAD^+^, was capable of significantly attenuating the H_2_O_2_-induced decrease in the intracellular ATP levels of PC12 cells (Fig. 2D).

### M-MSNs@NAD^+^ can Restore Both the Intracellular NAD^+^ and ATP Levels of H_2_O_2_-treated Primary Astrocyte Cultures

**Figure 3:**
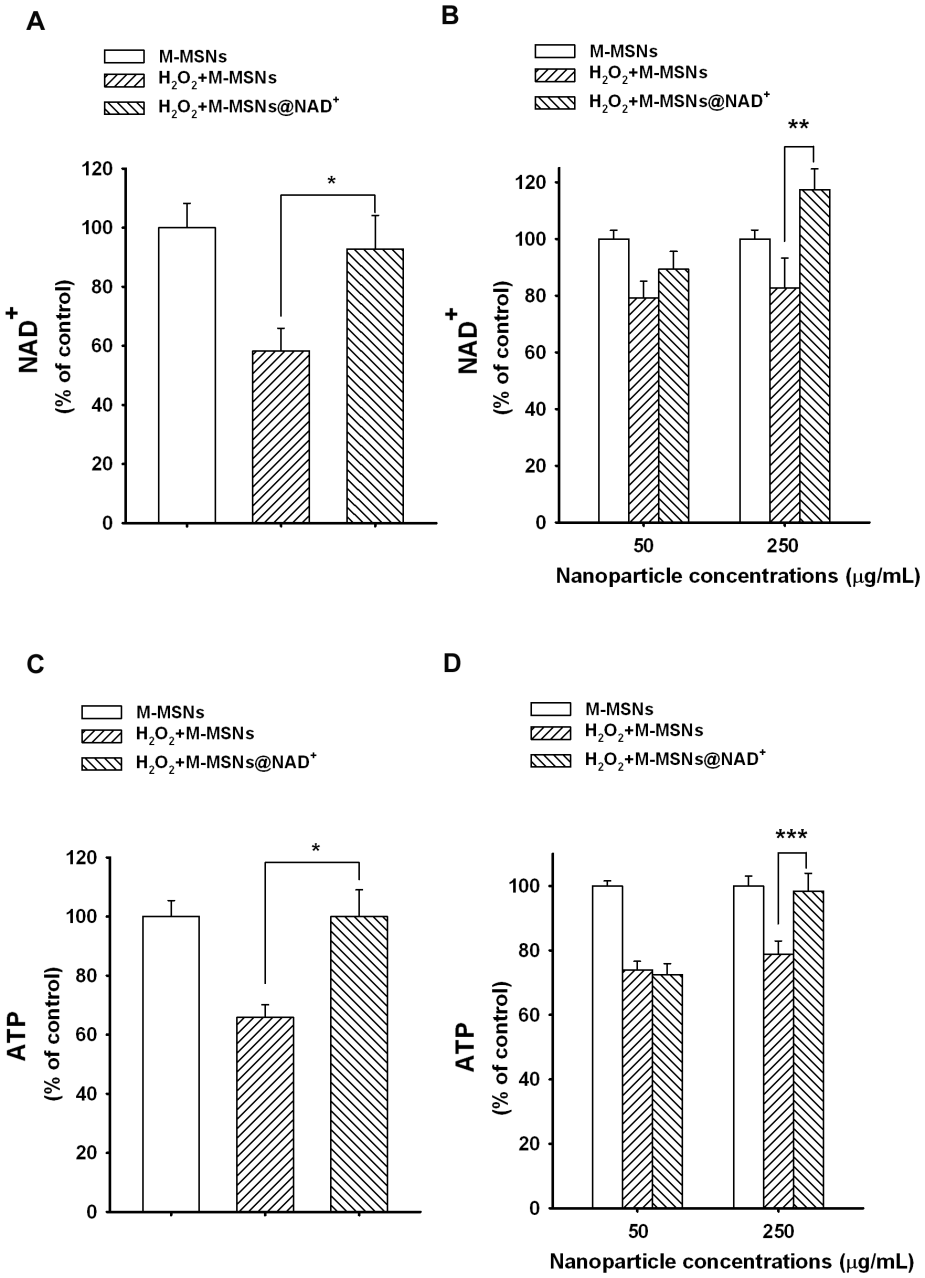
M-MSNs@NAD^+^ can significantly increase the intracellular NAD^+^ and ATP levels of H_2_O_2_-treated Astrocytes. (A) Treatment of the cells with 1 mM H_2_O_2_ led to a significant decrease in the intracellular NAD^+^ levels of astrocytes, which was prevented by post-treatment of the cells with 50 µg/mL M-MSNs@NAD^+^ for 3 hrs. N = 14. Data were collected from four independent experiments. *, *p*<0.05. (B) Assessed at 6 hours after nanoparticle post-treatment, 250 µg/mL M-MSNs@NAD^+^, but not 50 µg/mL M-MSNs@NAD^+^, was capable of preventing the H_2_O_2_-induced decrease in the intracellular NAD^+^ levels of astrocytes. N = 9∼12. Data were collected from three independent experiments. **, *p*<0.01. (C) Treatment of the cells with 1 mM H_2_O_2_ also led to a significant decrease in the intracellular ATP levels of astrocytes, which was also prevented by post-treatment of the cells with 50 µg/mL M-MSNs@NAD^+^ for 3 hrs. N = 22–23. Data were collected from three independent experiments. *, *p*<0.05. (D) Assessed at 6 hours after nanoparticles post-treatment, 250 µg/mL M-MSNs@NAD^+^, but not 50 µg/mL M-MSNs@NAD^+^, was capable of preventing the H_2_O_2_-induced decrease in the intracellular ATP levels of astrocytes. N = 13–16. Data were collected from four independent experiments. ***, *p*<0.001.

To further determine if the M-MSNs@NAD^+^ can enhance both the intracellular NAD^+^ levels and NAD^+^-dependent cellular functions in the cells exposed to oxidative stress, we also used H_2_O_2_-treated astrocyte cultures as a cellular model. Treatment of astrocytes with 1 mM H_2_O_2_ significantly decreased the intracellular NAD^+^ level of astrocytes (Fig. 3A), which was significantly improved by post-treatment of the cells with the 50 µg/mL M-MSNs@NAD^+^ for 3 hrs (Fig. 3A). We also determined the effects of M-MSNs@NAD^+^ on the intracellular NAD^+^ levels of H_2_O_2_-treated astrocytes at 6 hrs after the M-MSNs@NAD^+^ treatment, showing that 250 µg/mL M-MSNs@NAD^+^, but not 50 µg/mL M-MSNs@NAD^+^, was capable of significantly attenuating the H_2_O_2_-induced decrease in the intracellular NAD^+^ levels of astrocytes (Fig. 3B).

We also found that treatment of astrocytes with 1 mM H_2_O_2_ led to a significant decrease in the intracellular ATP level of astrocytes (Fig. 3C), which was significantly attenuated by post-treatment of the cells with 50 µg/mL M-MSNs@NAD^+^ for 3 hrs (Fig. 3C). We further determined the effects of M-MSNs@NAD^+^ on the intracellular ATP levels of H_2_O_2_-treated cell at 6 hrs after the M-MSNs@NAD^+^ treatment, showing that 250 µg/mL M-MSNs@NAD^+^, but not 50 µg/mL M-MSNs@NAD^+^, significantly attenuated the H_2_O_2_-induced decrease in the intracellular ATP levels of astrocytes (Fig. 3D).

### The Spontaneously Released NAD^+^ from M-MSNs@NAD^+^ was Insufficient to Affect the Intracellular NAD^+^ and ATP Levels of H_2_O_2_-treated PC12 Cells and Astrocyte Cultures

**Figure 4:**
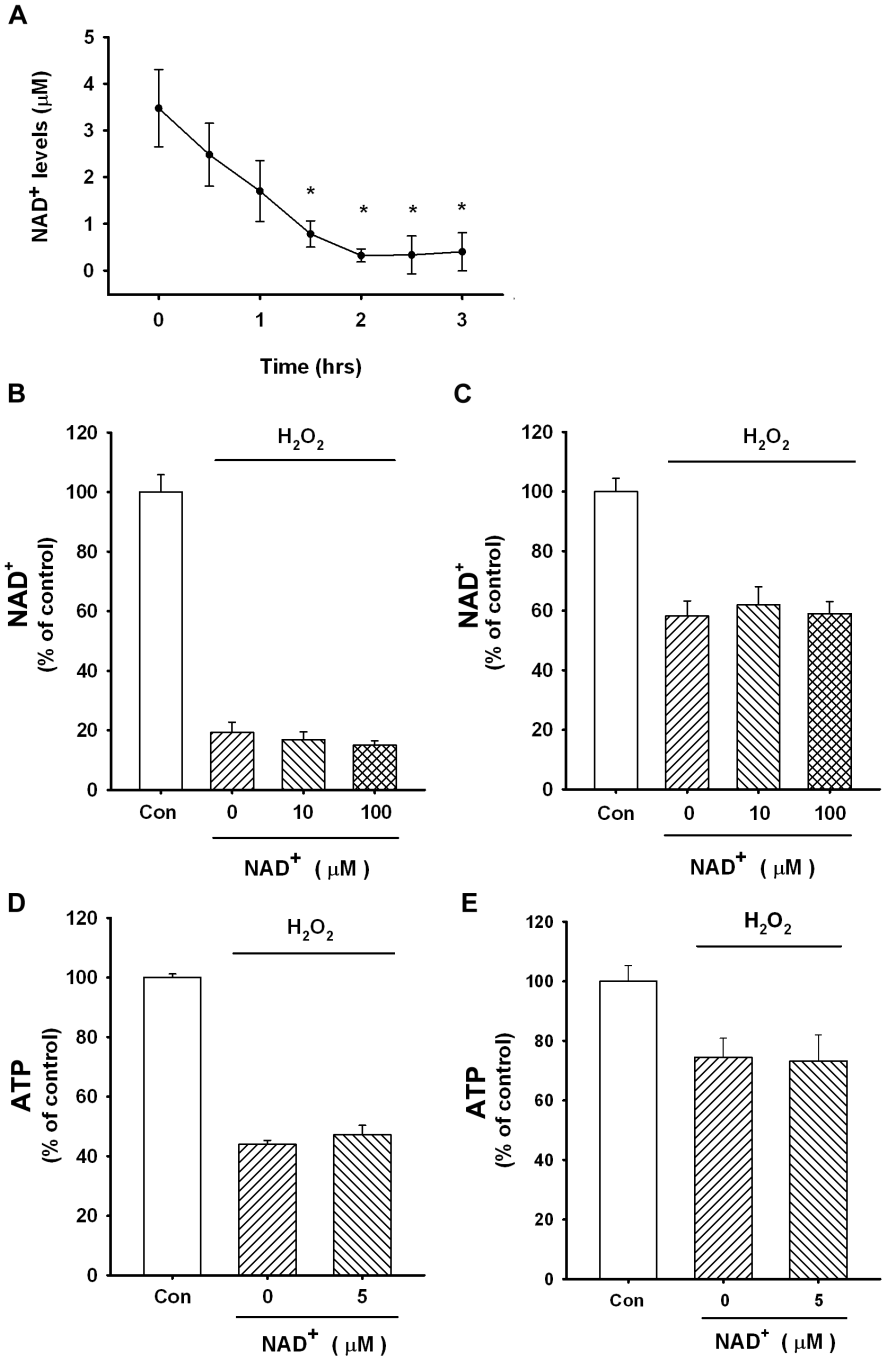
The NAD^+^ that is spontaneously released from M-MSNs@NAD^+^ can not increase the intracellular NAD^+^ and ATP levels in H_2_O_2_-treated PC12 cells. (A) The concentration of NAD^+^ that was spontaneously released from the 50 µg/mL M-MSNs@NAD^+^ into the media. NAD^+^ concentrations were measured by the recycling assay at various time points after the M-MSNs@NAD^+^ were added into the media. The peak NAD^+^ concentration was approximately 4 µM, which decreased gradually with time. N = 3. Data were collected from three independent experiments. *, *p*<0.05. (B) Treatment of PC12 cells with 10 ∼ 100 µM NAD^+^ could not increase the intracellular NAD^+^ levels in H_2_O_2_-treated PC12 cells. N = 12. Data were collected from three independent experiments. (C) When the astrocytes were treated with 10 ∼ 100 µM NAD^+^, no increase in the intracellular NAD^+^ was observed. N = 7. Data were collected from two independent experiments. (D) Treatment of PC12 cells with 5 µM NAD^+^ could not increase the intracellular ATP levels in H_2_O_2_-treated PC12 cells. N = 15∼19. Data were collected from four independent experiments. (E) Treatment of astrocytes with 5 µM NAD^+^ could not increase the intracellular ATP levels. N = 7∼8. Data were collected from two independent experiments.

Because previous studies have shown that high concentrations of NAD^+^ (1–10 mM) can led to significant protection of several types of cells exposed to genotoxic agents [2], [3], we used H_2_O_2_-treated PC12 cells and astrocyte cultures as cellular models to determine if the beneficial effects of the M-MSNs@NAD^+^ on the intracellular NAD^+^ and ATP levels in oxidative stress-exposed cells may result from the spontaneously released NAD^+^ from M-MSNs@NAD^+^ into cell media. NAD^+^ recycling assays were conducted to determine the spontaneously released NAD^+^ from the M-MSNs@NAD^+^ at various time points after the M-MSNs@NAD+ were added into the cell culture media. We found that the peak NAD^+^ concentration was approximately 4 µM, which decreased gradually with time (Fig. 4A). When PC12 cells (Fig. 4B) or the astrocytes (Fig. 4C) were treated with 10–100 µM NAD^+^ for 3 hrs, the intracellular NAD^+^ were not significantly increased in the H_2_O_2_-treated cells. When PC12 cells (Fig. 4D) or the astrocytes (Fig. 4E) were treated with 5 µM NAD^+^ for 3 hrs, the intracellular ATP were not significantly increased in the H_2_O_2_-treated cells. These results argue against the possibility that the beneficial effects of the M-MSNs@NAD^+^ on the intracellular NAD^+^ and ATP levels of H_2_O_2_-treated PC12 cells and astrocytes result from the spontaneously released NAD^+^ from the M-MSNs@NAD^+^.

### M-MSNs@NAD^+^ can Decrease the LDH Release from the H_2_O_2_-treated Astrocyte Cultures and PC12 Cells

**Figure 5:**
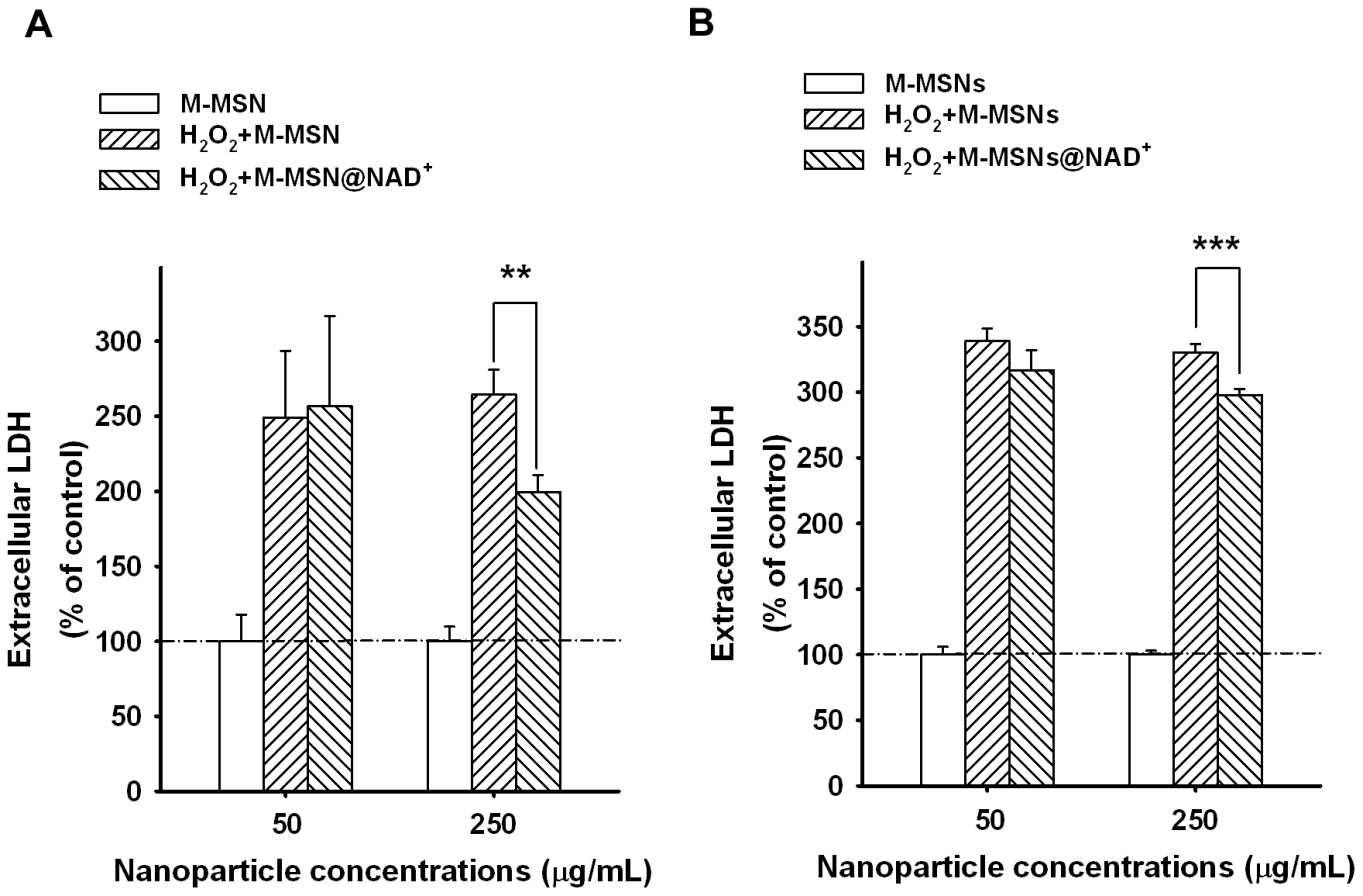
The M-MSNs@NAD^+^ can partially prevent H_2_O_2_-induced LDH release from both astrocyte cultures and PC12 cells. (A) Treatment of the cells with 1 mM H_2_O_2_ led to LDH release from astrocytes, which was significantly decreased by post-treatment of the cells with the 250 µg/ml M-MSNs@NAD^+^ for 24 hrs. N = 12 ∼ 21. Data were collected from three independent experiments. **, *p*<0.01. (B) Treatment of the PC12 cells with 1 mM H_2_O_2_ led to LDH release from the cells. Post-treatment of the cells with 250 µg/ml M-MSNs@NAD^+^ for 24 hrs produced a mild but statistically significant decrease in the H_2_O_2_-induced LDH release. N = 12. Data were collected from three independent experiments. ***, *p*<0.001.

We determined if M-MSNs@NAD^+^ may decrease H_2_O_2_-induce astrocyte death by measuring LDH release from the cells 24 hrs after H_2_O_2_ treatment. We found that post-treatment with 250 µg/mL M-MSNs@NAD^+^ significantly decreased the LDH release from the H_2_O_2_-treated astrocytes (Fig. 5A). We also determined if M-MSNs@NAD^+^ may decrease H_2_O_2_-induced PC12 cell death by measuring LDH release from the cells 24 hrs after H_2_O_2_ treatment. Our results showed that post-treatment of PC12 cells with 250 µg/mL M-MSNs@NAD^+^ can produce a mild but statistically significant reduction of the LDH release from the H_2_O_2_-treated PC12 cells (Fig. 5B).

### PARP Activation was Involved in H_2_O_2_-induced Impairments of Cellular Energy Metabolism and Cell Death of PC12 Cells

**Figure 6:**
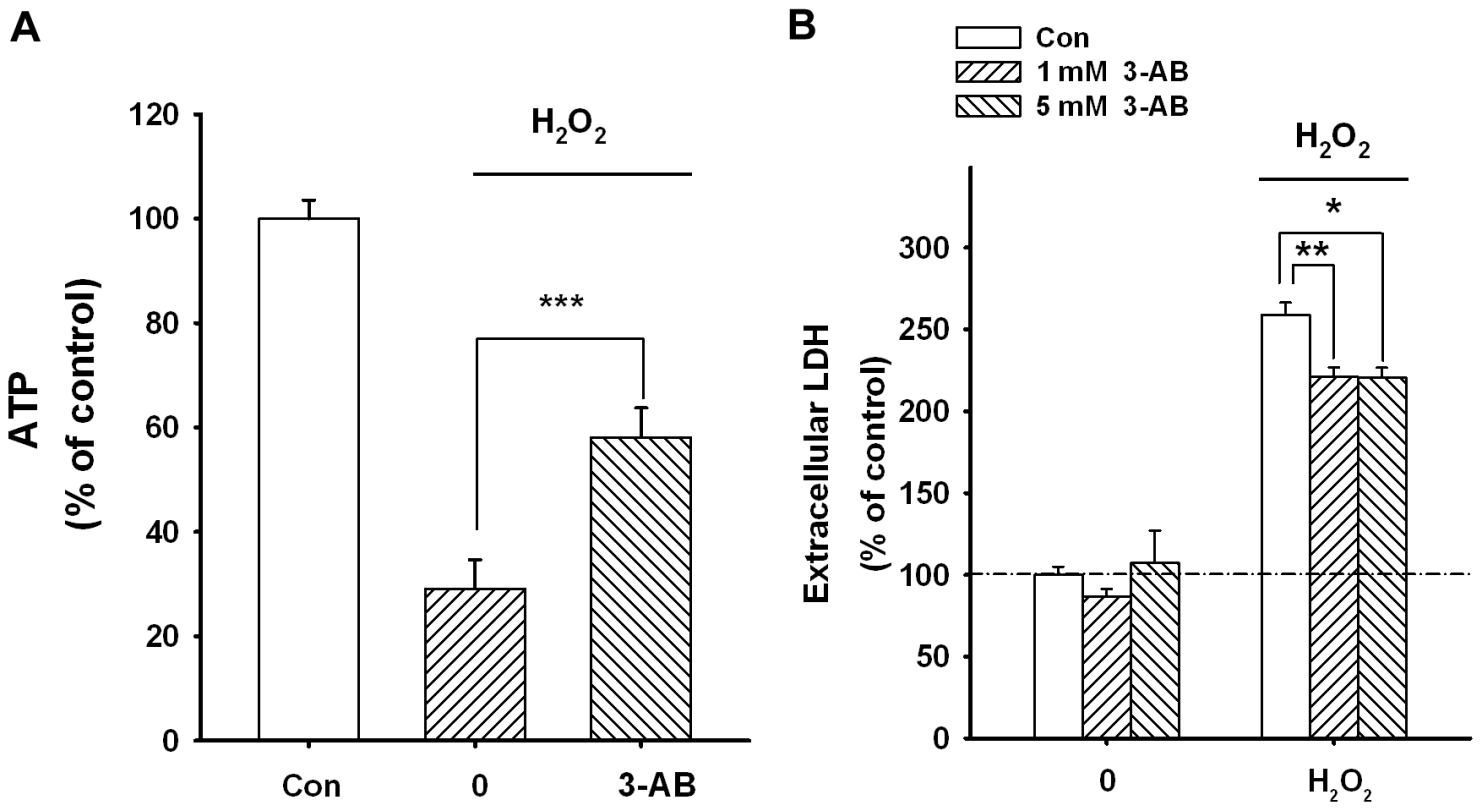
PARP activation was involved in H_2_O_2_-induced ATP decreases and cell death of PC12 cells. (A) PC12 cells were treated with DMEM containing both 1 mM H_2_O_2_ and 1 mM 3-aminobenzamide (3-AB) for 1 hr, and then the medium was removed and replaced with DMEM containing 1 mM 3-AB for 3 hrs. 3-AB treatment can attenuate the H_2_O_2_-induced ATP decrease in PC12 cells, indicating that PARP activation was involved in the detrimental effects of H_2_O_2_. N = 16. Data were collected from three independent experiments. ***, *p*<0.001. (B) Post-treatment of PC12 cells with 3-AB can produce a mild decrease in the H_2_O_2_-induced LDH release from PC12 cells. N = 12. Data were collected from three independent experiments. *, *p*<0.05; **, *p*<0.01.

Poly(ADP-ribose) polymerase (PARP), a major NAD^+^ consuming enzyme, appears to play significant roles in oxidative cell death under many conditions [1], [20]. Increasing evidence has suggested that NAD^+^ depletion may mediate PARP-induced cell death [1]. To determine the role of the PARP in H_2_O_2_-induced energy failures in our cellular model, we determined if 3-aminobenzamide (3-AB), a PARP inhibitor, can attenuated H_2_O_2_-induced damage of PC12 cells. We found that 3-AB significantly attenuated the H_2_O_2_-induced ATP decrease, indicating that PARP activation plays a role in the detrimental effects of H_2_O_2_ on the intracellular ATP levels (Fig. 6A). We also found that 3-AB can produce a mild but statistically significant reduction of the LDH release from the H_2_O_2_-treated PC12 cells (Fig. 6B), suggesting that PARP activation contributes to the detrimental effects of H_2_O_2_ on cell survival (Fig. 6B).

## Discussion

The major observations of our current study include: First, M-MSNs can be effectively loaded with NAD^+^; second, M-MSNs@NAD^+^ can effectively restore the intracellular NAD^+^ and ATP concentrations in both H_2_O_2_-treated astrocytes and H_2_O_2_-treated PC12 cells, which does not appear to result from the NAD^+^ that was spontaneously released from the M-MSNs@NAD^+^ into the media; and third, M-MSNs@NAD^+^ can also partially prevent the LDH release from H_2_O_2_-treated astrocytes and PC12 cells. Collectively, our study has suggested the first approach that can effectively deliver NAD^+^ into cells to produce cytoprotective effects. Our current study has also provided the first direct evidence indicating that NAD^+^ depletion mediates oxidative stress-induced ATP depletion, which is a key oxidative stress-induced pathological change.

NAD^+^ and NAD^+^-dependent enzymes mediate a variety of biological processes, including energy metabolism, mitochondrial functions, aging, and cell death [1]. It has also been found that NAD^+^ treatment can decrease the death of primary cultures of neurons, astrocytes, and myocytes exposed to oxidative stress [2] and DNA alkylating agents [3]. It appears to be increasingly important to elucidate the mechanisms underlying the roles of intracellular NAD^+^ in cell death and biological functions.

Most current cell culture studies investigate the roles of intracellular NAD^+^ on cell survival and biological functions by directly adding NAD^+^ into cell culture media [18]. However, there are two major problems that are associated with the usage of extracellullarly applied NAD^+^: First, 1 mM or higher concentrations of NAD^+^ are needed to produce significant protective effects of intracellular NAD^+^ and ATP levels; and second, there are several NAD^+^-dependent ecto-zymes, such as CD38 and mono(ADP-ribosyl) transferases on plasma membranes [7]. Both CD38 and mono(ADP-ribosyl) transferases may produce significant biological effects by consuming extracellular NAD^+^: CD38 is a multifunctional enzyme that can generate cyclic ADP-ribose (cADPR), a major endogenous agonist of ryanodine receptors on ER membranes, by using NAD^+^ as a substrate [21]. Mono(ADP-ribosyl) transferases on plasma membranes catalyze the mono-(ADP-ribosyl)ation of proteins such as P2X_7_ receptors by using NAD^+^ as a substrate. It has been reported that mono-(ADP-ribosyl)ation of P2X_7_ receptors can lead to opening of the receptors in such cell types as T regulatory cells leading to apoptosis of the cells [7]. Therefore, the extracellularly administered NAD^+^ may produce its effects partially by interacting with the ecto-enzymes, which would prevent solid elucidation of the roles of intracellular NAD^+^ in biological functions.

A previous study by delivering NADase into cell neurons indicated that NAD^+^ depletion is sufficient to induce such pathological changes as nuclear translocation of apoptosis-inducing factor (AIF) [18]. However, the study has not completely excluded the possibility that the extracellularly added NAD^+^ produces its effects partially by interacting with the NAD^+^-dependent ecto-enzymes. It was also reported that liposomes were used to carry NAD^+^ into cells to determine the roles of NAD^+^ in oxidative stress-induced tissue injury [22]. However, the major limitation of the liposome-based approach is the low efficacy of NAD^+^ delivery: This approach can not improve the intracellular NAD^+^ levels in oxidative stress-exposed cells by more than 20% [22]. Collectively, novel approaches that can effectively deliver NAD^+^ into cells are critically needed, which would greatly enhance our capacity to elucidate the biological functions of intracellular NAD^+^, and to enhance the potential of clinical applications of NAD^+^.

Because nanoparticles have shown great potential as multi-functional drug carriers, in this study we determine the effects of NAD^+^-carrying nanoparticles in delivering NAD^+^ into cells. Several lines of our current study have suggested that the M-MSNs can effectively carry NAD^+^ into both PC12 cells and primary astrocyte cultures: First, treatment of either primary astrocyte cultures or PC12 cells with M-MSNs@NAD^+^ can effectively restore the intracellular NAD^+^ in the H_2_O_2_-treated cells; second, treatment of either primary astrocyte cultures or PC12 cells with M-MSNs@NAD^+^ can also restore the intracellular ATP concentrations in the H_2_O_2_-treated cells; and third, our study has excluded the possibility that the beneficial effects of the M-MSNs@NAD^+^ result from the NAD^+^ that was spontaneously released from the M-MSNs@NAD^+^ into the media: The levels of NAD^+^ that is released from the M-MSNs@NAD^+^ into the media are below 5 µM, while our study showed that extracellular NAD^+^ at 5 µM can not increase the intracellular NAD^+^ and ATP levels in H_2_O_2_-treated PC12 cells. Collectively, our study has suggested the first approach that can effectively delivery NAD^+^ into cells.

Our current study has also provided the first direct evidence indicating that NAD^+^ depletion plays a key role in H_2_O_2_-induced ATP depletion. Oxidative stress plays critical roles in a number of diseases such as brain ischemia and Alzheimer’s disease [23], [24]. Impairments of energy metabolism is also one of the key pathological changes in multiple diseases [24]. It is of great theoretical and therapeutic significance to expose the relationships among oxidative stress, intracellular NAD^+^ levels and intracellular ATP levels. Our current study has shown that restoration of intracellular NAD^+^ levels can lead to marked recovery of intracellular ATP levels in both H_2_O_2_-treated astrocytes and H_2_O_2_-treated PC12 cells. Because our study has excluded the possibility that the effects of the M-MSNs@NAD^+^ result from the NAD^+^ that was spontaneously released from the M-MSNs@NAD^+^ into the media, our study has provided first direct evidence demonstrating a critical role of NAD^+^ depletion in H_2_O_2_-induced ATP depletion. This finding could significantly improve our understanding on the mechanisms underlying oxidative cell injury.

In our study we found that 50 µg/mL M-MSNs@NAD^+^ can prevent H_2_O_2_-induced ATP and NAD^+^ depletion at 3 hours after the M-MSNs@NAD^+^ treatment, but not at 6 hours after the treatment. In contrast, 250 µg/mL M-MSNs@NAD^+^ can prevent H_2_O_2_-induced ATP and NAD^+^ depletion at both 3 and 6 hours after the M-MSNs@NAD^+^ treatment. The detailed mechanisms underlying these observations are unclear at present. One possible explanation for these observations is as follows: H_2_O_2_-induced PARP-1 overactivation lasts for several hours [25]. Thus, while 50 µg/mL M-MSNs@NAD^+^ was capable of repleting the NAD^+^ in the H_2_O_2_-treated cells at 3 hours after the M-MSNs@NAD^+^ treatment, 50 µg/mL M-MSNs@NAD^+^ may have lower capacity than that required for repleting the NAD^+^ in the H_2_O_2_-treated cells at 6 hours after the M-MSNs@NAD^+^ treatment.

It is well established that different cell types may have different responses to extracellular stimuli. For example, glutamate can induce excitotoxic injury of neurons, but not astrocytes. Therefore, in studies that determine if drug carriers can effectively carry drugs into cells, it is beneficial for the value of the studies to use more than one cell type. Therefore, in the current study we used both primary astrocyte cultures and PC12 cell lines – a widely used cell line – to determine if the M-MSNs@NAD^+^ may effectively carry NAD^+^ into cells. Our study has indicated that M-MSNs@NAD^+^ can effectively deliver NAD^+^ into the cells to increase the intracellular ATP and NAD^+^ levels in both of the cell types. These observations have collectively suggested that M-MSNs@NAD^+^ is a novel and effective carrier of NAD^+^.

Our current study showed that treatment of the cells with 250 µg/mL MMSNs@NAD^+^ can nearly completely prevent the H_2_O_2_-induced decreases in both intracellular NAD^+^ and ATP levels in astrocytes and PC12 cells. In contrast, 250 µg/mL MMSNs@NAD^+^ can only partially prevent the H_2_O_2_-induced increases in extracellular LDH release from astrocytes and PC12 cells. Based on previous studies, our current study and theoretical considerations, it does not appear to be surprising that there are differences between the MMSNs@NAD^+^-produced protection on the intracellular NAD^+^ and ATP levels and the MMSNs@NAD^+^-produced protection on the death of the cells: 1) Previous studies have reported that such PARP inhibitors as 3-aminobenzamide and benzamide could nearly completely prevent the H_2_O_2_-induced NAD^+^ decrease in astrocyte cultures and PC12 cells, while the PARP inhibitors only partially decreased the H_2_O_2_-induced cell death in both of the cell types [26], [27]. Our current study (Fig. 6) has also shown that 3-AB treatment led to an over one-fold increase in the intracellular ATP levels in the H_2_O_2_-treated PC12 cells, while the 3-AB treatment produced only a mild protection against H_2_O_2_-evoked LDH release from the cells; and 2) the following is the theoretical considerations: Since it is well established that H_2_O_2_ can impair multiple cellular components such as membrane lipids, mitochondria, and DNA [28], prevention of decreases in intracellular NAD^+^ and ATP levels may be insufficient to produce complete protection on cell death. Our current study using NAD^+^-carrying nanoparticles have further suggested that, at least for certain cell types, complete protection of H_2_O_2_-induced decreases in intracellular ATP and NAD^+^ levels does not necessarily lead to complete protection on H_2_O_2_-induced cell death.

## Conclusions

Our study has suggested a novel approach for delivering NAD^+^ into cells, which establishes an important approach to solidly elucidate the roles of intracellular NAD^+^ in biological functions and cell death. Our study has also suggested a new NAD^+^-delivery approach for potential applications of NAD^+^ for treating such neurological diseases as brain ischemia and head trauma. Moreover, our study has provided the first direct evidence demonstrating a critical role of NAD^+^ depletion in H_2_O_2_-induced ATP depletion.
